# Perceived related humor in the classroom, student–teacher relationship quality, and engagement: Individual differences in sense of humor among students

**DOI:** 10.1016/j.heliyon.2023.e13035

**Published:** 2023-01-14

**Authors:** Lu'mu, Ani Cahyadi, Muhammad Ramli, Ruslan, Hendryadi

**Affiliations:** a Faculty of Engineering, Universitas Negeri Makassar, Makassar, Indonesia; b Tarbiyah and Teacher Training Faculty, Universitas Islam Negeri Antasari, Banjarmasin, Indonesia; c Sekolah Tinggi Ilmu Ekonomi Indonesia Jakarta, Jakarta, Indonesia

**Keywords:** Perceived related humor, Student–teacher relationship quality, Student engagement, Sense of humor, TSRQ, IHPT

## Abstract

This study explored the effect of humor on teacher–student relationship quality (TSRQ) and student engagement by uncovering the mediating role of TSRQ and the moderating role of individual differences (personal sense of humor). Data were collected using a cross-sectional time-lag approach with 2 phases; 367 students participated. The hypotheses were tested with a moderated mediation model. Perceived humor was positively related to TSRQ and student engagement. The results also confirmed the mediating role of TSRQ; a sense of humor positively moderated the relationship between perceived related humor and TSRQ, as well as perceived related humor and student engagement. The present study uncovers the relationship between humor and relationship quality in learning settings. Moreover, our study provides the first empirical data on the mediating effects of TSRQ on perceived related humor and student engagement. It also reveals the role of individual differences (sense of humor) in the proposed model.

## 1 Introduction

The effectiveness of humor used by instructors in the classroom on students' related-learning behavior has attracted the attention of many researchers in the last two decades at the elementary to higher education levels [1–3]. In general, humor is defined as a message that aims to cause laughter and entertain through incongruous meanings [4]. Researchers have focused on several issues, including the type of humor [5,6] and humor's effect on learning [2,[7], [8], [9]]. In the context of learning, humorous interludes can create a more pleasant atmosphere in the classroom [10], reducing stress and depression [11]. Despite mixed results on the effect of the use of humor by teachers in class on students' attitudes and behavior [4], most studies confirmed its essential role in creating a positive learning atmosphere [10,12]. The present study focuses on understanding how the humor used by instructors can encourage positive student attitudes and behaviors, including improved teacher–student relationship quality (TSRQ) and students at the university level.

Existing works agree that not all types of humor can be used effectively in the classroom [5,6,8]. For example, Ref. [6] classified types of humor (appropriate and inappropriate) based on students' perceptions, which are key to how humor works. Similarly, using the instructional humor processing theory (IHPT) perspective [5], developed a typology revealing why humorous stimuli may or may not work. Frymier et al.‘s work has become a guide for subsequent studies examining the effects of humor in learning settings. In this study, we used “related humor” or “appropriate humor”, which has previously been documented for student engagement [2,[7], [8], [9]].

The main aim of this study is to determine when and how humor used by instructors can positively affect student engagement in higher education's online learning environment. We were interested in studying student learning engagement because this issue is a challenging and increasingly complex undertaking in online learning during the COVID-19 pandemic [13]. Although existing studies have examined the relationship between humor and student engagement [2,7,8], except for Ref. [2], student engagement has yet to be examined in the online learning context. Hence, our study adds to the latest empirical evidence regarding the relationship between humor in learning and student engagement in online learning.

Next, we proposed TSRQ as an outcome of perceived humor that has not been explored. According to Refs. [12,14], humor behavior is a communication skill closely related to efforts to forge meaningful connections and foster a supportive learning environment. However, in terms of relationship quality, existing studies have focused more on the work environment [15–17]. In addition, as a substitute model to comprehend the process of student engagement, we tested the process model by identifying the intermediate function of TSRQ in the relationship between perceived related humor. Hence, our study offers new empirical evidence of the effectiveness of humor in improving relationship quality in online learning modes. Furthermore, a high level of relationship quality between instructors and students increases the participation and engagement of college students.

**Fig. 1:**
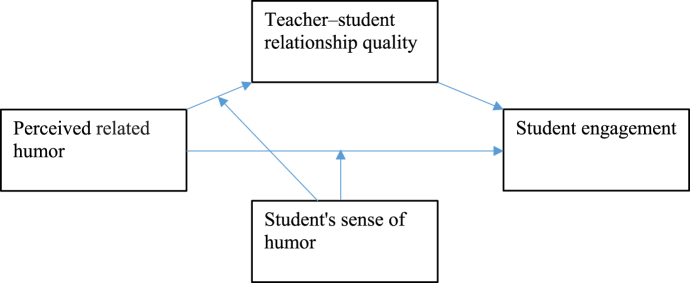
Research model.

Finally, we identified how individual differences (students' sense of humor) caused them to respond differently to perceived related humor. Previously, researchers focused on gender, culture, and race as potential factors that cause differences in perceptions of humor in educational settings [18–20]; instead, we placed students' sense of humor. There are two reasons why a students' sense of humor is essential in this model: first, prior studies are more interested in examining the teacher's sense of humor [8], and sense of humor from the perspective of the recipients of the message (students) is rarely studied. Second, existing studies focused on personal traits to investigate the effectiveness of humor interventions [11] on happiness, depression, and well-being. In the present study, we sought to determine whether a humorous learning intervention would be suitable for both humorous and non-humorous students, especially concerning relationship quality and engagement (see Fig. 1).

### 1.1 Theoretical background and hypotheses development

We used the relational process model of humor (RPMH) [15,21] to explain how humor can build good relationships between senders (instructors) and recipients (students), which can eliminate hierarchies, raise voices, and signal togetherness [17]. Drawing on the RPMH, we proposed that the humor initiated by an instructor in a classroom can improve the quality of the relationship between the instructor and their students. In this study, we framed a high-quality relationship between instructors and students that can be created through the instructor's humorous behavior, which forms a pleasant learning atmosphere in the classroom.

The IHPT was developed by Wanzer et al. [6] to explain teacher humor behavior in the classroom and its effect on students' attitudes and learning behavior. Particularly, the IHPT explains why humor in the classroom can positively and negatively impact students. The theory is based on a series of processes, such as incongruity, which refers to whether students recognize the humorous message as congruous. Thus, when students resolve the incongruity, a humorous message is conveyed, and students laugh. Furthermore, perceived humor is responded to either positively or negatively. Both positive (appropriate humor) and negative (inappropriate humor) effects, affect students’ motivation to engage with humorous messages differently. The IHPT divides different types of humor (appropriate/inappropriate, related/unrelated humor) based on their effects on students' cognitive and affective states. In other words, not all humor is helpful in learning activities [10]. The IHPT has been extensively used in research to look at the connection between humor and related learning behavior [3,8,22,23].

### 1.2 Perceived related humor and teacher–student relationship quality

Humor is a form of entertainment intended to bring laughter [4,8]. It can be used to break the silence and rigidity of a serious atmosphere to create a positive learning atmosphere and improve group cohesion, creativity, and student motivation [4,6,10]. Through humor, instructors can break the ice in the classroom, reducing the stress, fatigue, and boredom of students. It has the primary function of building relationships [21]; thus, it is unsurprising that the use of humor by teachers in the classroom can improve the relationship between teachers and students.

Theoretically, the relationship between humor and relationship quality can be explained using RPMH [15,15,21,21]. This theory argues that good relationships can be created through humor that can break silences, and hierarchies, raise voices and signal togetherness between instructors and students [17]. Moreover, the link between humor and relationship quality has previously been documented by researchers in the workplace context [15,17,24,25]. For example, Ref. [15] used a social exchange perspective to explain how leader humor can create positive emotions and subsequently influence employee citizenship behavior. Leader humor is also positively associated with leader–member relationship quality and job satisfaction [25]; it also decreases cynicism [24]. In an employee setting in China, Tan et al. found that leader humor was related to perceived organizational support, engagement, and job crafting [16]. A more recent study [17] found that leader humor can improve relationship quality between subordinate leaders and the subsequent effect on the upward voice. Considering the empirical evidence, we argue that humor creates a more intimate atmosphere and interaction between instructors and students and consequently affects the quality of their relationship.

H1 Perceived related humor is positively associated with TSRQ.

### 1.3 Perceived related humor and student engagement

The most popular engagement concept comes from Ref. [26] as a combination of vigor, dedication, and absorption; describes students' positive emotional state and continuity when participating in the learning process [28]. Similarly, Fredricks et al. (2004) defined student engagement as a combination of three components: emotional, cognitive, and behavioral. The emotional component is students' positive and negative attitudes related to learning activities. In this case, students may respond negatively to the lack of social interaction in the online classroom or their relationships with peers and teachers. Next is the cognitive component, referring to how students' attitudes respond to the learning methods provided by the teacher, including pedagogic abilities and mastery of technology to trigger student creativity and self-controlled their responsibility. Third, the behavioral aspect of engagement refers to how students behave in learning activities, such as participating in class, completing assignments on time, and showing perseverance in their studies [29–31].

The relationship between students' perceived instructor humor and learning engagement has been documented. Humor a way for instructors to attract the attention of the class, and researchers have long tried to explain the effects of teachers’ humor on the learning atmosphere [10,12]. Wanzer et al. [6] emphasized that appropriate humor can have a positive effect, especially on student attention. Other studies [2,32] found different variations in the type of humor with students' emotional engagement, and that “related humor” can reduce boredom. Meanwhile, Ref. [3] supported the IHPT, where students' cognitive learning improved through perceived instructor humor. Using the IHPT and the empirical evidence above, we suspected that perceived humor relates positively to learning engagement.

H2 Perceived related humor is positively associated with student engagement.

### 1.4 Teacher–student relationship quality and student engagement

Learning is a process of interaction between teachers and students; it is undeniable that the ability of teachers to build relationships with their students is the key to effective learning in the classroom [33,34,35]. This study postulated teacher–student relationship quality as a learning and personal resource in developing student engagement in learning activities. Relationship quality between teachers and students has the characteristics of mutual trust and respect [36] derived from social interactions that contribute to intrinsic motivation and students' positive perceptions of their teachers [37,38]. Recently [31], used the positive and negative learning (PNL) model framework [30] to explain the relationship between loneliness and student engagement. Their findings confirm that loneliness as an emotional resource plays a significant role in influencing student engagement.

Prior research found various positive effects of high-quality teacher–student relationships on student attitudes and behavior, including student engagement [20,35,39,40], student achievement [40,41,42,43], prosocial behavior, social skills [43,44], and self-concept [45]. A High-quality relationships between teachers and students are characterized by mutual trust and respect, fairness, openness to student voices, and collaboration, which are characteristics of effective teachers who drive successful classrooms [35]. Furthermore, Thornberg and colleagues found that student engagement can be increased through a high level of teacher–student relationship quality [35]. Using theoretical arguments and empirical evidence described earlier, we propose that student engagement can be increased through social interaction in the form of quality relationships between instructors and students in online learning activities.

H3 TSRQ is positively associated with student engagement.

We also propose an indirect relationship between perceived related humor and student engagement via relationship quality. First, the relationship between humor and relationship quality has previously been documented [[15], [16], [17],^24^,^25^]. Second, previous studies also supported the link between TSRQ and student engagement [20,35,39], so we also propose that relationship quality will mediate the link between perceived related humor and student engagement.

H4 Relationship quality is mediated by perceived related humor and student engagement.

### 1.5 Moderating effect of students’ sense of humor

A sense of humor is considered by some researchers to be a personal characteristic or personality trait [46], and others have explored personality differences relating to one's sense of humor [11,47,48]. In this study, we replicated the research of Wellenzohn et al. [49] on the moderating role of the sense of humor of participants in interventions to reduce depression and increase happiness through humor-based positive psychology interventions. Thus, we expected students with higher levels of humor to benefit more from perceptions related to humor in the classroom. In other words, the effect of humor perception on relationship quality and student engagement will be higher in students with a high sense of humor compared to a low one.

H5 Students' sense of humor will moderate the effect of perceived related humor on TSRQ.

H6 Students' sense of humor will moderate the effect of perceived related humor on student engagement.

## 2 Methods

### 2.1 Participants and procedure

This study involved three universities that were willing to be involved voluntarily. Two teaching staff members represented each university as collaborators based on the university leadership's recommendation and the collaborators' approval. Collaborators are selected based on the criteria of having a high and low sense of humor based on the leader's assessment. This approach is taken to obtain variability in the sample of instructors at the same university and the same subject. The collaborators in the study acted as representatives from each university to make it easier for the research team to access student data.

Students voluntarily filled out the questionnaire, which was confidential and anonymous, and did not receive any compensation for this activity. In addition, because the data collection process was carried out in two stages, students were free to stop at any phase to ensure that participation was voluntary. They were fully informed about the data collection process.

Data collection was carried out in two phases: at time 1 (T1), students were asked to fill in demographic information and personal ratings about their sense of humor. Meanwhile, the instructors were asked to answer questions related to their humor habits in class. In this phase, respondents' email address was recorded for the next phase of the data collection process. In this phase, we try to examine the similarity of responses between lecturers in assessing themselves and students' perceptions of their lecturers (an explanation of technical detail in the measurement section). Time 2 (T2) was conducted in the middle of the semester; participants were asked via email to answer instructor humor and learning engagement questions. A total of 367 undergraduate students (245 males and 122 females) were involved in this study. The participants were 18–27 years old, and the mean age was 20 (see Table 1).

**Table 1:** Participant characteristics and data consistency of perceived instructor humor.

Group	ICC(1)	Mann-Whitney	Gender		Age		
		Test (p-value)	Male	Female	<21 yrs	21–25 yrs	>25 yrs
Group 1	0.60	0.22	62	11	35	26	12
Group 2	0.52	0.89	60	8	54	10	4
Group 3	0.67	0.69	65	13	66	11	1
Group 4	0.52	0.13	8	36	28	12	4
Group 5	0.56	0.35	25	21	35	9	2
Group 6	0.68	0.24	25	33	58	0	0
Total			245	122	276	68	23

### 2.2 Measures

The perceived related humor measure was adapted from Ref. [5] to measure students' attitudes toward their instructor's humor behavior in class. We made minor adjustments for seven items. An example item is “Your instructor uses humor related to course material.” All items were responded to on a 5-point Likert scale ranging from 1 (nothing/never) to 5 (absolutely/always). Because the data on instructor humor were obtained from two sources (students and instructors), to ensure the alignment of responses between students in the group, the interclass correlation (ICC) and Mann-Whitney *U* test were employed to examine the differences in student and instructor responses. Table 1 shows that the ICC(1) value obtained was in the range of 0.52–0.68, meeting the reliability standard between students in the group [50]. In addition, the results of the Mann-Whitney *U* test also showed no significant difference in the average student-instructor response related to the instructor's humorous behavior in class. The Cronbach's alpha for the scale was .93.

TSRQ was measured using three items adapted from Ref. [51]. A minor revision was carried out on each item to describe the close relationship between students. For example, “Does your instructor understand your problems and needs?” and “Does your instructor recognize your potential?” All items were rated on a 5-point Likert scale according to context, ranging from 1 (nothing/never) to 5 (absolutely/always). The Cronbach's alpha for the scale was 0.87.

The student learning engagement measure was adapted from the short Utrecht Work Engagement Scale (UWES-9) developed by Ref. [52]. A minor revision has been applied to adjust the location where the word “workplace” is replaced with “online class.” Previous studies have similarly employed this method [13,28,53], to adapt the context of workplace engagement to classroom learning settings. The Cronbach's alpha for the scale was 0.89 and met the recommended internal consistency standard [54].

The short scale of sense of humor scale [55] was used to assess students' self-described sense of humor. The scale consists of 6 items and three dimensions (cognitive, social, and affective). For example, “Do you easily recognize, as a sign of humor, an allusion or a slight change of emphasis?“and “People who make humor irritate me because they clearly want others to laugh.” Students rated all items on a 4-point scale depending on the context provided (1 = very easily/it is not true, 4 = very hard/yes indeed). The Cronbach's alpha for the scale 0.73 (see Table 2).

**Table 2:** Descriptive statistics, correlation matrix, and internal consistency.

No	Variable	*Cronbach α*	*Mean*	*S.D*	1	2	3	4	5	6
1	Gender	–	–	–	1					
2	Age	–	–	–	−.08	1				
3	PRH	.93	2.99	.76	−.03	.03	1			
4	TSRQ	.87	3.36	.80	−.05	−.03	.19**	1		
5	ENG	.89	3.49	.83	.03	−.03	.29**	.40**	1	
6	SH	.73	3.13	.77	.08	−.04	.19**	.25**	−.02	1

Note: PRH = perceived related humor, TSRQ = teacher–student relationship quality, ENG = engagement, SH = sense of humor, **p < .01.

Control variables: We added two control variables (gender and age) based on the consideration that both are related to a personal sense of humor [19,56] as well as the perceived quality of the relationship between students and teachers [35,57]. Both variables are coded: female (1), male (2), <20 yo (1), 20–25 yo (2), and above 25 yo (3).

### 2.3 Statistical analysis

Data analysis in this study used the individual level, considering that the number of groups was insufficient to analyze at the group level [58,59]. First, we used descriptive and correlation analyses to provide an initial overview of the data. Second, a moderation mediation analysis was conducted to examine the effect of perceived instructor humor on students' perceptions of TSRQ and student engagement. Also, the moderating role of a sense of humor was included in the model. We used the process macro for SPSS [60], model 8. The hypotheses were tested by evaluating the p-values and using a bootstrap method with 5000 resamples, based on Hayes's (2017) recommendation.

## 3 Results

First, we checked the means and standard deviations of the study variables. As shown in Table 2, students' perceptions of the instructor's humor behavior in class were moderate: (M = 2.99, SD = 0.76), TSRQ (M = 3.36, SD = 0.80), student engagement (M = 3.49, SD = 0.83), and sense of humor (M = 3.13, SD = 0.77). Next, the bivariate correlation between variables showed a positive relationship between perceived related humor and TSRQ (r = 0.19, p < .01) and student engagement (r = 0.29, p < .01). TSRQ was shown to be positively related to student engagement (r = 0.40, p < .01).

The results provided empirical support for all hypotheses regarding the moderation mediation analyses to test the study hypotheses (see Table 3). After controlling gender and age, the results found that perceived related humor was positively related to TSRQ (β = 0.14, p < .05) and student engagement (β = 0.25, p < .01). TSRQ was positively related to student engagement (β = 0.38, p < .01). Thus, H1 to H3 were supported.

**Table 3:** Summary of regression results.

	Coefficient	SE	t	P	LLCI	ULCI
Gender	−.10	.08	−1.16	.25	−.27	.07
Age	−.04	.07	−.63	.53	−.18	.09
PRH	.14	.05	2.62	.01	.03	.25
SH	.23	.05	4.28	.00	.12	.33
Interaction	.15	.06	2.24	.03	.02	.27
Gender	.12	.08	1.58	.12	−.03	.27
Age	−.03	.06	−.42	.67	−.15	.10
PRH	.25	.05	5.07	.00	.15	.34
TRSQ	.38	.05	7.97	.00	.29	.47
SH	−.18	.05	−3.58	.00	−.27	−.08
Interaction	.22	.06	3.64	.00	.10	.33

Table 4 shows the results of the mediation moderation analysis. The indirect effect of perceived related humor on student engagement via TSRQ was 0.05, with a 95% confidence interval (LLCI 0.01; ULCI 0.10). Since zero was not included in the interval (LL and UL), the indirect effect was statistically significant. Thus, H4 was supported. The direct and indirect effects of perceived instructor humor on student engagement were also significant via TSRQ, suggesting the plausibility of partial mediation by these parameters.

**Table 4:** Unstandardized coefficients for the moderation and mediation analyses.

Conditional indirect effects	*Coeff.*	*BootSE*	*BootLLCI*	*BootULCI*
*PRH > TRSQ > ENG*	.05	.02	.01	.10
Index of moderated mediation	.06	.03	.01	.11
Conditional effects of perceived related humor on TRSQ based on sense of humor				
*Low sense of humor (-1 SD)*	.03	.08	−.12	.18
*High sense of humor (+ 1 SD)*	.25^a^	.07	.12	.39
Conditional effects of perceived related humor on student engagement based on sense of humor				
*Low sense of humor (-1 SD)*	.08	.07	−.05	.22
*High sense of humor (+ 1 SD)*	.41^a^	.06	.29	.54

a p < .01.

The moderating effect of a sense of humor was tested based on the value of the interaction. A sense of humor was shown to significantly moderate the relationship between perceived related humor and TSRQ (β = 0.15, p < .05). Similarly, a sense of humor also moderated the relationship between perceived related humor and student engagement (β = .22, p < .01). Thus, H5 and H6 were supported.

**Fig. 2:**
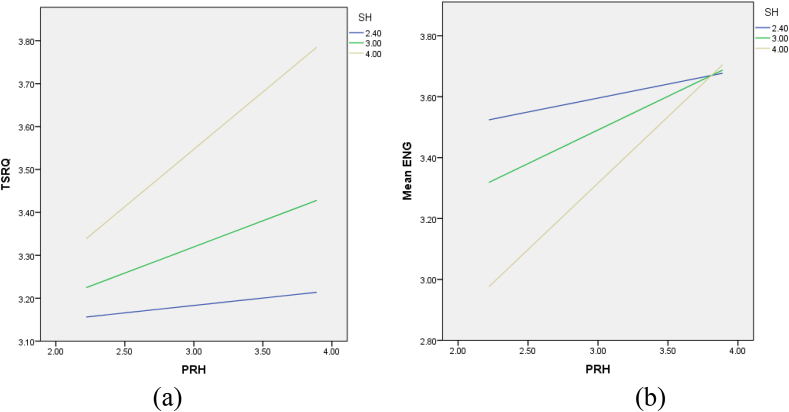
Conditional effect of perceived related humor on (a) TSRQ and (b) student engagement; based on students' sense of humor.

Furthermore, the simple slope effect, as shown in Table 4 and Fig. 2(a and b), provided information about the direct effect of perceived related humor on TSRQ and student engagement based on the value of a sense of humor. The simple slope of TSRQ based on the value of a sense of humor was only significant at a high level: low level (β = 0.03, p > .05) and high level (β = 0.25, p < .01). The research findings also show that a sense of humor is essential as a potential moderator. The effect of perceived humor related to student engagement is statistically significant only when the sense of humor is high (β = 0.41, p < .01), and not significant when it was at a low level (β = 0.08, p > .05).

## 4 Discussion

The use of humor by instructors in classroom learning activities is a topic of great interest to researchers in the world of education. Apart from the inconsistency of the results on the relationship of the humor-learning outcome due to differences in the methodology used by previous researchers [1], research in the last two decades has reached some general agreement regarding the positive effects of humor used by teachers on learning outcomes. In the context of how humor can be effective, Frymier et al. [5] developed a typology revealing why humorous stimuli may or may not work, known as instructional humor processing theory.

This study provides new empirical evidence on the relationships between perceived related humor, TSRQ, student engagement, and sense of humor. This study supports IHPT and RPMH in explaining students' responses to instructor's humorous behavior in the classroom. The results confirmed that perceived related humor is positively related to TSRQ and student engagement, and student engagement is positively related to student engagement. TSRQ was also proven to mediate the relationship between perceived related humor and student engagement in the mediation model. Finally, the moderation model was partially proven, where a sense of humor moderated the relationship between perceived related humor and TSRQ and student engagement. In summary, the findings of our study make significant contributions to the literature on humor in the learning environment, its effectiveness in improving the quality of teacher–student relationships, and student engagement.

The first hypothesis predicted that perceived related humor would positively correlate with TSRQ; this hypothesis was supported. Previously, the relationship between humor and relationship quality was studied in the workplace [[15], [16], [17],^24^,^25^], in the context of relationships between leaders and subordinates. Our findings provide some preliminary support for the relationship between perceived humor and relationship quality in the context of education. Furthermore, the findings showed that students interpreted the instructor's perceived related humor as an effective communication signal to build close relationships. These results are also consistent with the RPMH [15,21]; humorous behavior acts as a driver to build good relationships between senders (instructors) and recipients (students).

The second hypothesis predicted that perceived related humor would be positively correlated with student engagement. This hypothesis was supported; this finding supports previous studies [2,7,8]. It can be stated that instructors who have a humorous teaching style in explaining learning materials draw the attention of students so that they engage in learning activities. In this study, we argued that the instructor's ability to create humorous schemes related to learning materials shows a good understanding of the material and extensive experience in the field.

Similarly, the third hypothesis predicted that a high-quality teacher–student relationship would increase student engagement. This hypothesis was supported and is consistent with previous studies [20,35,39,40]. Moreover, the fourth hypothesis predicted that TSRQ would mediate the relationship between perceived related humor and student engagement. This study provides the first data on the mediating effects of TSRQ on perceived related humor and student engagement. Our findings supported the RMPH model by considering the continued effect of TSRQ on student engagement. Humor can create effective communication and quality relationships between teachers and students, as an after-effect of being a driver of student engagement in the classroom [20,35,39,40]. Thus, both perceived related humor and TSRQ are effective learning resources to push students' learning engagement to a higher level. The fifth and sixth hypotheses predicted the role of a sense of humor as a moderator of the effect of perceived related humor on TSRQ and student engagement.

The results of this study were consistent with those of Wellenzohn et al. [49], who examined the role of sense of humor but in a different context; namely, the effectiveness of an intervention through humor to reduce depression and increase happiness. Taken together, the results in this study provide preliminary empirical evidence regarding the effect of individual differences (sense of humor) on the effectiveness of teachers' humorous behavior in the classroom relating to student attitudes and behavior (TSRQ and student engagement). In other words, students' sense of humor plays an essential role as a boundary condition in the relationship between perceived related humor, TSRQ, and student engagement. The results provide the critical information that a greater level of students’ sense of humor promotes the effectiveness of humor in the classroom in the context of TSRQ and student engagement.

The results of our study also have several practical implications, particularly for the education sector. First, universities need to train teachers in managing classes, including the ability to create a pleasant atmosphere through humor appropriate to the context of the material. Second, because humor behavior is closely related to personality [11,47,48], higher education administrators, especially human resources management personnel, must be more stringent in selecting prospective instructors by considering andragogic competence and communication. Finally, considering that the effectiveness of learning through the instructor's humor behavior is dependent on the recipients—namely, the sense of humor of students—we suggest that instructors study the classroom atmosphere at the beginning of the semester. The first meeting can be the starting point for the instructor to understand the class so that the learning methods can be modified accordingly.

### 4.1 Limitations and future research suggestions

Despite some critical theoretical and practical implications, the present study has several limitations. First, methodologically, the model tested used cross-sectional data, because of which the causal relationship between variables could not be ascertained. Even though the present study applies a time lag in the data collection process, we still recommend that future studies consider a longitudinal or mixed experimental design approach to obtain better causality claims. Second, our study focused on related humor and did not consider other types of humor [6]. Future research should use other types of humor to predict TSRQ and student engagement. Third, the participants in this study were undergraduate students in two provincial capitals in Indonesia. Future studies should consider expanding the sample, taking samples from different regions to investigate the model reported in this study. Finally, student engagement in our study is measured in the context of learning in online classes. Future studies can explore information on student engagement in various curricular and co-curricular activities outside the classroom to get different perspectives.
