# Supplementary material for: Perceived related humor in the classroom, student–teacher relationship quality, and engagement: Individual differences in sense of humor among students
Source: Heliyon. 2023 Jan 14;9(1):e13035. doi: 10.1016/j.heliyon.2023.e13035 (PMC9868540; doi:10.1016/j.heliyon.2023.e13035)
Supplement: Multimedia component 1 [file mmc1.docx]

**Research Instrument**

**Perceived related humor**

For instructor

1. Use humor related to course material.
2. Use funny props to illustrate a concept or as an example.
3. Tell a joke related to course content.
4. Tell a humorous story related to course content
5. Performs or acts out course material to illustrate concepts.
6. Facilitates student role-play exercises to illustrate course content.
7. Uses language in creative and funny ways to describe course material

*For student*

1. Your instructor uses humor related to course material.
2. Your instructor use funny props to illustrate a concept or as an example.
3. Your instructor tell a joke related to course content.
4. Your instructor tell a humorous story related to course content
5. Your instructor performs or acts out course material to illustrate concepts.
6. Your instructor facilitates student role-play exercises to illustrate course content.
7. Your instructor uses language in creative and funny ways to describe course material

**Teacher-student relationship quality (TRSQ)**

1. How would you characterize your relationship with your teachers?
2. Does your instructor understand your problems and needs?
3. Does your instructor recognize your potential?

**Student engagement.**

1. I am enthusiastic about my online class
2. My online class give me more inspiration
3. I can't wait to join the online class
4. I feel happy when I am learning intensely
5. I don't procrastinate doing schoolwork
6. Time feels fast when taking online classes
7. I enjoy the fun of online learning
8. Time feels fast when taking online classes
9. I enjoy the fun of online learning

**The sense of humor**

1. Do you easily recognize, as a sign of humor, an allusion or a slight change of emphasis?
2. Would it be easy for you to find something comical, witty or humorous in most situations?
3. People who try to be funny are very irresponsible and should not be taken into account.
4. People who make humor irritate me because they clearly want others to laugh
5. Would you say that you have a lot of fun reasons during a normal day?
6. It’s my impression that those who try to be funny do it to hide theirs lack of self-confidence?
